# Experimental and Theoretical Study of Cyclic Amine Catalysed Urethane Formation

**DOI:** 10.3390/polym14142859

**Published:** 2022-07-13

**Authors:** Hadeer Q. Waleed, Dániel Pecsmány, Marcell Csécsi, László Farkas, Béla Viskolcz, Zsolt Fejes, Béla Fiser

**Affiliations:** 1 Institute of Chemistry, University of Miskolc, 3515 Miskolc-Egyetemváros, Hungary; kemhader@uni-miskolc.hu (H.Q.W.); pecsmany.daniel@gmail.com (D.P.); csecsi.marcell2@gmail.com (M.C.); kemviskolcz@uni-miskolc.hu (B.V.); 2 Higher Education and Industrial Cooperation Centre, University of Miskolc, 3515 Miskolc-Egyetemváros, Hungary; 3 Wanhua-BorsodChem Zrt, Bolyai tér q. 1, 3700 Kazincbarcika, Hungary; laszlo.farkas@borsodchem.eu; 4 Ferenc Rákóczi II Transcarpathian Hungarian College of Higher Education, 90200 Beregszász, Ukraine

**Keywords:** amine catalysts, kinetics, catalyst free, composite method, ab initio

## Abstract

The alcoholysis of phenyl isocyanate (PhNCO) using stoichiometric butan-1-ol (BuOH) in acetonitrile in the presence of different cyclic amine catalysts was examined using a combined kinetic and mechanistic approach. The molecular mechanism of urethane formation without and in the presence of cyclic amine catalysts was studied using the G3MP2BHandHLYP composite method in combination with the SMD implicit solvent model. It was found that the energetics of the model reaction significantly decreased in the presence of catalysts. The computed and measured thermodynamic properties were in good agreement with each other. The results prove that amine catalysts are important in urethane synthesis. Based on the previous and current results, the design of new catalysts will be possible in the near future.

## 1. Introduction

Polyurethanes (PUs) represent an important class of polymers that have found widespread use in many products in our daily life. PU is a macromolecular polymer including several urethane linkages that are formed by the reaction between -NCO groups (isocyanate) and -OH groups (polyol). Isocyanates and polyols are responsible for the different properties of PU products such as flexibility and hardness. Due to the fact of their excellent properties, such as high-temperature resistance, good flexibility, and excellent mechanical properties, the application range of PU is becoming wider and wider including adhesives, coatings, and rubbers [1,2,3,4]. It has been one of the most important automotive seating materials since 1960. Nowadays, seating foams are usually produced in a cold cure process, which generally requires that the polyol components be premixed with the crucial additives of the foam (i.e., catalysts, surfactants, blowing agent, crosslinker, fillers, and pigments). During manufacturing, the premix should be mixed with the isocyanate component in the calculated ratio and dosed into the preheated mould (~50–60 °C) [5,6]. Synthesizing PU from isocyanates and alcohol under industrial conditions requires a combination of catalysts which will expedite the chemical reactions [7,8]. All in all, catalysts can be considered as one of the most important components of the reaction system besides the starting materials [9]. The resulting foam quality is strongly dependent on the two primary catalytic reactions of polyurethane foaming. The first is the gelling reaction, where the chains are growing, and the second is the foaming reaction, where CO_2_ is inflating the material, which leads to the cellular structure of the foam. These reactions must be balanced for the proper quality of the final product and depends on the used catalysts [10]. Tertiary-amine-containing structures are usually used as catalysts in polyurethane reactions [11]. Therefore, much research aimed at studying their catalytic effect on polyurethane formation by using both theoretical and kinetic methods [11,12,13]. Thus, the catalytic process and selectivity in many cases are well understood [14]. In a previous work, the catalytic effect of triethylamine on polythiourethane synthesis was studied using kinetic measurements, and the results showed enhancement in the reaction rate with the increase in the catalyst concentration [15]. Computational studies have also been carried out to describe the catalytic process [16,17]. The catalytic effect of different amine catalysts on urethane formation has been studied using the BHandHLYP density functional theory (DFT) method in combination with the 6-31G(d) basis set and the G3MP2BHandHLYP composite method [11,12]. The results showed that by adding a catalyst to the system, the activation energy was significantly reduced, and urethane formation was promoted [11]. Despite all the efforts to understand polyurethane synthesis, there is still room for improvement in terms of the fine details of the reactions.

**Figure 1:**
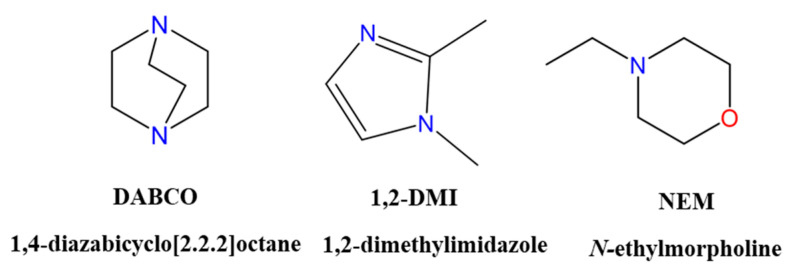
Chemical structures of the studied catalysts.

Three different cyclic amine catalysts (Figure 1) were studied, and their catalytic activity in urethane synthesis was compared by using kinetic measurements.

Furthermore, the catalytic reactions were also examined by using computational tools to determine the step-by-step mechanisms with and without catalysts and to compare the reaction pathways (Figure 1). Catalyst design and development will be possible by describing the catalytic urethane formation at the molecular level.

## 2. Methods

### 2.1. Materials and Methods

#### Materials

The preparation of methyl phenylcarbamate and butyl phenylcarbamate was performed by reacting phenyl isocyanate (PhNCO, ≥99%, Acros Organics, Geel, Belgium) with the corresponding alcohol, methanol (MeOH, HPLC grade, VWR Chemicals, Debrecen, Hungary) and butan-1-ol (BuOH, ≥99%, VWR Chemicals, Debrecen, Hungary) in excess, respectively, and acetonitrile (ACN, ≥99%, VWR Chemicals, Debrecen, Hungary) was used as a solvent. To achieve a low water content, BuOH (≥99%, VWR Chemicals, Debrecen, Hungary) and ACN (≥99%, VWR Chemicals, Debrecen, Hungary) were stored in over 20% (m/V) activated molecular sieves (3 Å, beads, VWR Chemicals, Debrecen, Hungary) for at least two days [18]. The products were purified by flash column chromatography (hexane/ethyl acetate) on silica. The studied catalysts were 1,4-diazabicyclo[2.2.2]octane (DABCO, 98%, Alfa Aesar, Kandel, Germany); 1,2-dimethylimidazole (1,2-DMI, 98%, Alfa Aesar, Kandel, Germany); *N*-ethylmorpholine (NEM, 98%, Alfa Aesar, Kandel, Germany).

### 2.2. Kinetic Experiments

Stock solutions of 0.5 M PhNCO, 0.5 M BuOH, and 0.01 M catalyst in ACN were prepared in 25 mL volumetric flasks. Meanwhile, to prepare the latter, the catalyst was pipetted from a 0.05 M stock solution (ACN) to achieve a more precise measurement. Therefore, to start the reaction, 5.0 mL PhNCO solution and 5.0 mL BuOH and catalyst from the prethermostated stock solutions were pipetted into a prethermostated empty headspace glass vial using a mechanical pipette, and then the vial was capped. The experiments were conducted at 303, 313, 323, and 333 K. A Hamilton syringe was used to withdraw 10 µL of a sample from the reaction mixture, and it was mixed into a chromatography vial containing 990 µL MeOH in order to quench the reaction. Unreacted isocyanate forms methyl phenylcarbamate spontaneously with a very high excess of MeOH. The quenched samples were then subjected to HPLC analysis.

### 2.3. Analysis Method

**Figure 2:**
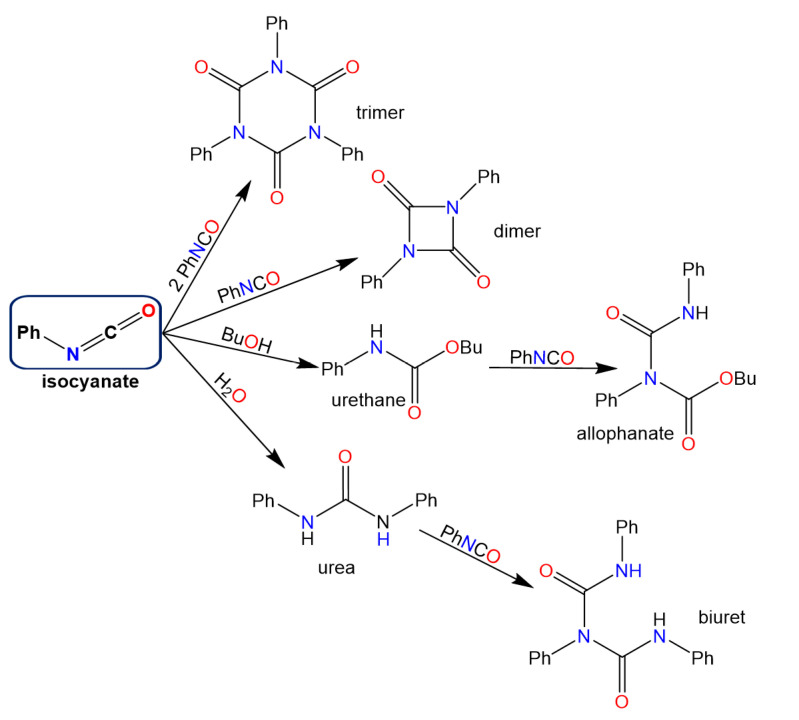
Possible products of the phenyl isocyanate (PhNCO)/butan-1-ol (BuOH) reaction system. Low residual water content giving rise to urea formation originates from the solvent.

Shimadzu HPLC (Shimadzu Corporation, Kyoto, Japan) equipped with LC-20AD pumps, an SIL-20AC autosampler, a DGU-20A3R degassing unit, a CTO-20A column oven, and an SPD-M20A photodiode array detector were used for the analysis of the quenched and diluted samples. For the separation, a SunShell C8 column (2.6 µm, 150 × 3.0 mm; ChromaNik Technologies Inc., Osaka, Japan) thermostated at 40 °C was used. The injection volume was 1 µL. The eluent was ACN:H_2_O with a gradient as follows: 45% ACN at 0 min, 45–75% ACN at 0–3 min, 75% ACN at 3–5 min, 75–45% ACN at 5–7 min, and, finally, 45% ACN at 7–10 min. The flow rate was 0.5 mL/min. The reaction products (Figure 2) were quantified at 246 nm (Figure S1).

### 2.4. Theoretical Method

Calculations were performed by using the Gaussian 09 program package [19]. The BHandHLYP/6-31G(d) level of theory in combination with the SMD solvent model (acetonitrile, *ε*_r_ = 35.688), which was previously proved to be efficient to study catalytic urethane formation reactions [11], was applied for geometry optimizations. Furthermore, the G3MP2BHandHLYP composite calculation scheme was used to improve the accuracy of the calculations [11,20,21]. The reaction mechanisms without and in the presence of catalysts were examined, and the corresponding structures (i.e., reactants, intermediates, products, and transition states) were located. Moreover, intrinsic reaction coordinate (IRC) calculations were carried out [22] to ensure that the located transition states connected the desired reactants and products. The potential energy surface (PES) of the reaction mechanisms were analysed. Proton affinities (PAs) were also determined, and the computed and experimental values were compared [23].

## 3. Results and Discussion

### 3.1. Results of the Kinetic Experiments

Determination of the rate constants (*k*) at different temperatures was conducted by plotting the reciprocal isocyanate concentration (1/[NCO]) against time (*t*) and applying a linear fitting using the kinetic equation for second-order reactions (Equation (1)). The isocyanate concentration ([NCO]) was equal to the concentration of methyl phenylcarbamate generated by quenching with methanol. [NCO]_0_ was 0.25 M, which was the starting isocyanate concentration during the experiments.

(1) $$\frac{1}{\left[\mathrm{NCO}\right]}=k\times t+\frac{1}{{\left[\mathrm{NCO}\right]}_{0}}$$

**Figure 3:**
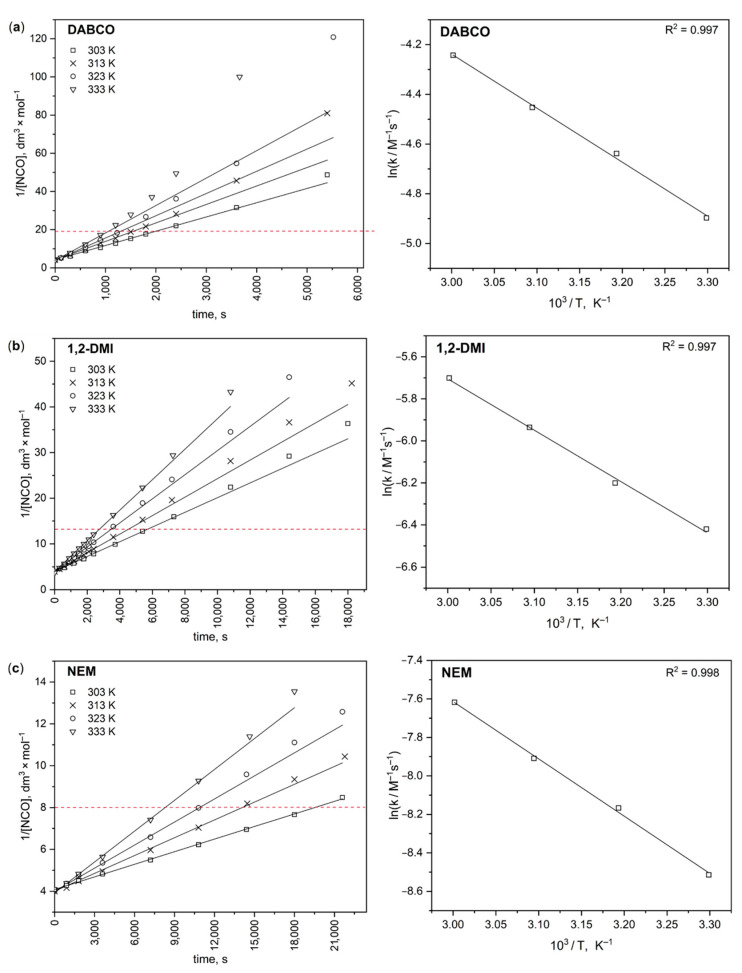
(**a**) Experimental kinetic curve (left) and Arrhenius plot (right) for urethane formation in the presence of 1,4-diazabicyclo[2.2.2]octane (DABCO) catalyst. (**b**) Experimental kinetic curve (left) and Arrhenius plot (right) for urethane formation in the presence of 1,2-dimethylimidazole (1,2-DMI) catalyst. (**c**) Experimental kinetic curve (left) and Arrhenius plot (right) for urethane formation in the presence of *N*-ethylmorpholine (NEM) catalyst. Data points up to approximately 50–80% PhNCO conversion (indicated by the red, dotted lines) were used for fitting and rate constant determination.

The experimental kinetic curves for the PhNCO–BuOH reaction in the presence of DABCO, 1,2-DMI, and NEM (Figure 3) were plotted. Second-order kinetics was used to describe the reactions. Data points from these time segments of the reactions were used for linear fitting and calculating the rate constants (Table 1). It can be seen that the reactions showed a positive deviation from first-order kinetics at PhNCO conversion values of approximately 50%, 70%, and 80% for NEM, 1,2-DMI, and DABCO, respectively. As side products were formed only in low concentrations, this phenomenon might be attributed to the autocatalytic effect of the urethane product [24]. As the reactions proceeded, the degree of this deviation became increasingly pronounced, and it will be more obvious at higher reaction temperatures.

A significantly similar tendency in the kinetic data can be seen during the reaction of hexamethylene diisocyanate and diethylene glycol catalysed by an amine–manganese complex [25] and in the reaction of PhNCO with 2-ethylhexanol (and also with water) in the presence of DABCO [26]. The reaction rate constants (Table 1) show that NEM had the lowest catalytic effect, while in the presence of DABCO, the highest rate was experienced. In terms of catalytic activity, 1,2-DMI was located between the other two catalysts. In addition to the desired urethane product, PhNCO dimer and allophanate were also detected in low concentrations. Less than 0.4% and 0.5% of PhNCO converted into PhNCO dimer (<5 × 10^−4^ M) and allophanate (<6 × 10^−4^ M), respectively. Furthermore, urea was also formed to a slightly higher extent, but the maximum concentration (3 × 10^−3^ M) at the end of the reactions corresponded to only a 2.4% PhNCO conversion.

### 3.2. Results of the Theoretical Calculations

**Scheme 1:**
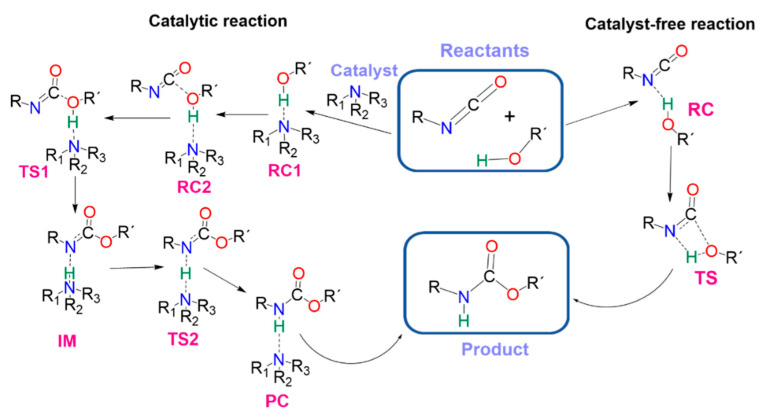
General reaction mechanisms for urethane formation without and in the presence of amine catalysts. RC—reactant complex; TS—transition state; IM—intermediate; PC—product complex.

In line with the kinetic experiments, the catalytic activity of the studied cyclic amine catalysts (Figure 1) in urethane formation (i.e., PhNCO and BuOH reaction) were compared using computational tools (Scheme 1) [27,28,29,30] to understand the reactions from a mechanistic point of view. The energetic and structural features of the PhNCO and BuOH reaction were described. Based on a recent study [11], a general reaction mechanism (Scheme 1) was applied for urethane formation in the case of a catalyst-free system and in the presence of catalysts.

It can be seen that the catalyst-free reaction goes through a concerted step (Scheme 1). First, the reactant complex (RC), including PhNCO and BuOH, is formed. In the studied case, a hydrogen bond between the hydroxyl group of BuOH and the nitrogen of the NCO group of PhNCO formed with a corresponding distance of 2.182 Å (Figure S2). In the next step, a transition state (TS) developed in which a proton transfer between the BuOH hydroxyl group and the nitrogen of the NCO group took place (1.387 Å), while a C-O bond was formed between the NCO’s carbon and the butan-1-ol’s oxygen (1.494 Å). The relative energy of the TS compared to the entrance channel was 119.1 kJ/mol (Table 2), and after this, the final urethane product (P) was reached.

**Figure 4:**
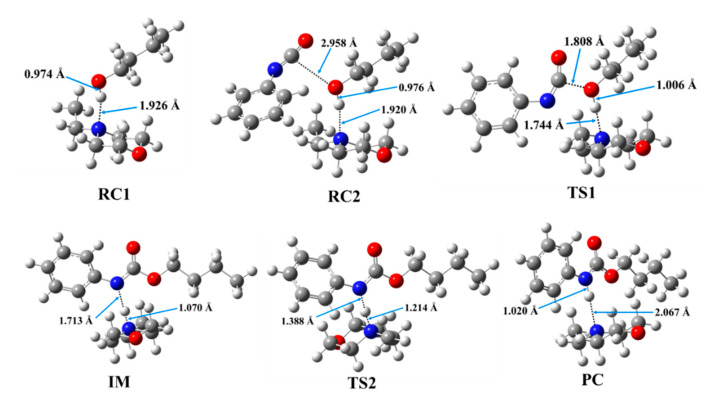
Optimised structures along the reaction pathway between phenyl isocyanate and butan-1-ol in the presence of *N*-ethylmorpholine (NEM) calculated at the BHandHLYP/6-31G(d) level of theory in acetonitrile at 298.15 K and 1 atm. RC—reactant complex; TS—transition state; IM—intermediate; PC—product complex.

The mechanism was also examined in the presence of catalysts (Scheme 1, Figure 4, Figures S3 and S4). It can be seen that additional structures were formed compared to the catalyst-free case.

As proton transfers are crucial during the reactions, proton affinity (PA) was computed for all unique nitrogen that were considered catalytically active within the cyclic amine catalysts (Figure 1, Table 3). The PAs of the catalytically active nitrogens were in a range of 973.2−1002.9 kJ/mol. The results showed that the difference between the calculated and data in the literature was 20.5 kJ/mol in the case of DABCO and 18.1 kJ/mol for 1,2-DMI. As the nitrogen of NEM had the lowest proton affinity (973.2 kJ/mol), after protonation, it was the most prone to donating its proton. Meanwhile, 1,2-DMI was the best proton acceptor, as it had the highest proton affinity (1002.9 kJ/mol).

During the industrial urethane synthesis, the catalyst was mixed into the polyol. Thus, in the presence of catalysts, the reaction was mimicked by the formation of the first complex (RC1) between the catalyst and the alcohol. The distance between the catalyst’s nitrogen and the hydroxyl hydrogen of butan-1-ol was in the range of 1.856 and 1.926 Å (Table 4, N-H *). Then, isocyanate was added to the system, which led to the formation of a trimolecular complex (RC2). In the case of RC2, an interaction occured between the oxygen of BuOH and the NCO group of the isocyanate, with the corresponding C-O distance being in the range of 2.958–3.073 Å, while a minor change in the length of the N-H * bond could be identified compared to the same interaction in RC1 (Table 4). 

**Figure 5:**
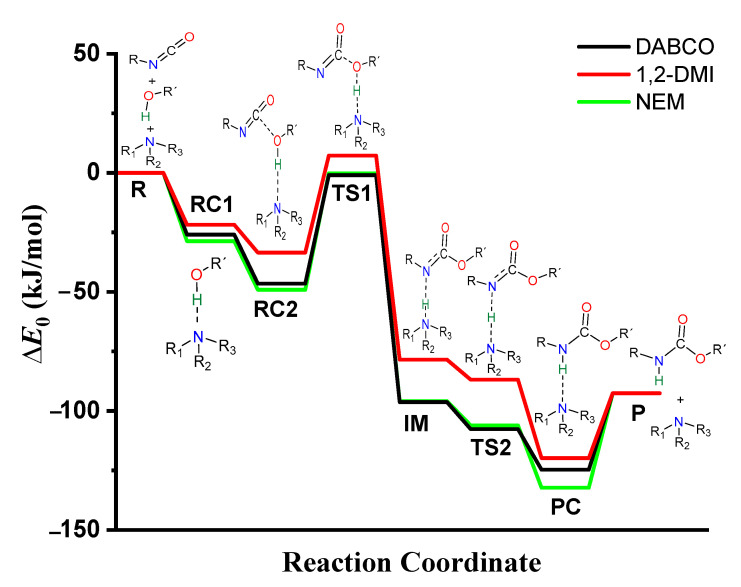
Energy profile (zero-point corrected, ΔE_0_) of the studied catalysed urethane formation reactions in the presence of 1,4-diazabicyclo[2.2.2]octane (DABCO), 1,2-dimethylimidazole (1,2-DMI), and *N*-ethylmorpholine (NEM) calculated at the G3PM2BHandHLYP level of theory in acetonitrile using the SMD implicit solvent model at 298.15 K and 1 atm.

The formed complexes (i.e., RC1 and RC2) were the most favoured in the case of NEM (Δ*E*_0_ = −28.7 and −49.1 kJ/mol, for RC1 and RC2, respectively), while in the presence of 1,2-DMI, they were the least stable (Δ*E*_0_ = −21.8 and −33.5 kJ/mol, for RC1 and RC2, respectively) compared to the other studied catalysts (Table 2 and Figure 5). As the catalytic reaction mechanism included proton transfer steps, the proton affinity of the catalytic nitrogen influenced the relative energy of the reaction steps (e.g., the reactant complex (RC1)), and by increasing the proton affinity, the corresponding thermodynamic property also changed (Figure S5). After the formation of the reactant complexes, the reaction continued with TS1 in which a proton transfer occurred from the hydrogen of the OH group to the nitrogen of the catalyst, and the corresponding distance between these groups significantly decreased to the range of 1.661–1.744 Å in the studied systems. The potential energy curve showed that in the presence of DABCO, the TS1 step had the lowest relative energy (Δ*E*_0_ = −0.9 kJ/mol) within the studied set of catalysts (Figure 5). The N=C=O group was bent activating the carbon for the formation of a new C–O bond, and this led to the formation of an intermediate (IM), where the distance between the hydrogen of the OH and the nitrogen of the catalyst significantly decreased, while a bond formed between the carbon of the N=C=O and the oxygen of the BuOH. The relative energy of the IM is lowest in the presence of DABCO (−96.2 kJ/mol). After the formation of the IM, the proton transfer occurred from the catalyst to complete the formation of the urethane bond. In this step, the N-H * increased, and the N-H ** decreased compared to the IM. The relative energy of TS2 was lowest in the presence of DABCO (Δ*E*_0_ = −107.6 kJ/mol) compared to the other two (i.e., 1,2-DMI and NEM) catalysts.

The penultimate step in the catalytic mechanism was the formation of the product complex (PC) in which a new bond formed between the hydrogen of the BuOH and the nitrogen of the N=C=O. The corresponding distance was significantly decreased, and it was in the range of 1.020–1.023 Å (Table 4, Figure 4, Figures S3 and S4). This led to the final step in this mechanism, namely, the separation of the catalyst from the product. The relative energy for the product was significantly reduced to −92.6 kJ/mol (Table 2 and Figure 5). It was found that the presence of the catalyst in urethane formation significantly changed the reaction mechanism compared to the catalyst-free case. There was a multi-step pathway to form the product. The presence of catalysts significantly reduced the relative energies, and the barrier height decreased (ΔΔ*E*_0_ > 110 kJ/mol) compared to the catalyst-free reaction. It can be seen that there was good agreement between the measured *E*_a_ values (i.e., 18.1 ± 0.7, 20.3 ± 0.8, and 24.8 ± 0.8 kJ/mol) and the corresponding calculated ones (i.e., 24.9, 29.1, and 28.5 kJ/mol) for the DABCO-, 1,2-DMI-, and NEM-catalysed reactions, respectively. It must be noted that the measured activation energy correlated with the energy difference between the TS1 and RC1 (Figure 5), as the first step in the experiment is to mix the catalyst into the alcohol. The highest difference was only 8.8 kJ/mol in the case of 1,2-DMI, while the lowest difference (3.7 kJ/mol) was experienced in the case of NEM. These results prove the validity of the proposed mechanism and verify the method selection as well.

## 4. Conclusions

The catalytic effect of cyclic amines on urethane formation was examined by using both experimental and computational tools. The phenyl isocyanate (PhNCO) and butan-1-ol (BuOH) reaction was used as the model system. Kinetic investigation of the alcoholysis of phenyl isocyanate (PhNCO) using stoichiometric butan-1-ol (BuOH) in acetonitrile was examined. A previously proposed general reaction mechanism was applied, and the thermochemical properties for the reaction without and in the presence of the catalysts were studied using density functional theory and composite methods. The measured activation energies for the studied catalysts (i.e., DABCO, 1,2-DMI, and NEM) were 18.1 ± 0.7, 20.3 ± 0.8, and 24.8 ± 0.8 kJ/mol, respectively. The corresponding calculated values were in good agreement with the measured ones, and the difference was only 6.8, 8.8, and 3.7 kJ/mol for DABCO, 1,2-DMI, and NEM, respectively, which proves the validity of the suggested mechanism and verifies the method selection as well. As the studied catalytic mechanism contains protonation steps, the proton affinities (PAs) of the catalytic nitrogens were also calculated. The proton affinity affected the relative energy of the corresponding reaction steps, and by increasing proton affinity, the Δ*E_0_* was also increased. The achieved result showed that computational tools can be used to describe similar systems in upcoming studies. Furthermore, based on these results, it will be possible to develop and design new catalysts in the future.

## Figures and Tables

**Table 1:** Initial reaction rate constants (*k*) for urethane formation in the presence of the studied catalysts, 1,4-diazabicyclo[2.2.2]octane (DABCO), 1,2-dimethylimidazole (1,2-DMI), and *N*-ethylmorpholine (NEM), at different temperatures, along with the corresponding Arrhenius activation energies (*E*_a_) and pre-exponential factors (*A*). The *E*_a_ and *A* values were obtained by the method of least squares.

Temperature, °C	DABCO	1,2-DMI	NEM
	*k* × 10^4^, M^−^^1^ s^−^^1^	*k* × 10^4^, M^−^^1^ s^−^^1^	*k* × 10^4^, M^−^^1^ s^−^^1^
30	74.7 ± 0.6	16.3 ± 0.1	2.01 ± 0.01
40	96.8 ± 0.8	20.3 ± 0.2	2.84 ± 0.03
50	116.4 ± 0.8	26.4 ± 0.3	3.68 ± 0.03
60	143.6 ± 3.1	33.4 ± 0.4	4.92 ± 0.06
***E_a_***, **kJ mol^−1^**	18.1 ± 0.7	20.3 ± 0.8	24.8 ± 0.8
***A***, **M^−1^ s^−1^**	9.7 ± 2.4	5.1 ± 1.5	3.7 ± 1.1

**Table 2:** Zero-point corrected relative energies (**Δ*E*_0_**), relative enthalpies (**Δ*H***), and Gibbs free energies (**Δ*G***) of the reaction between phenyl isocyanate and butan-1-ol in the presence of the studied catalysts, 1,4-diazabicyclo[2.2.2]octane (DABCO), 1,2-dimethylimidazole (1,2-DMI), and *N*-ethylmorpholine (NEM), calculated at the G3MP2BHandHLYP level of theory in acetonitrile using the SMD implicit solvent model at 298.15 K and 1 atm. R—reactant; RC—reactant complex; TS—transition state; IM—intermediate; PC—product complex; P—product.

	Δ*E*_0_ (kJ/mol)							
	R	RC1	RC2	TS1	IM	TS2	PC	P
**Cat.-free**	0.0	-	−11.2 *	119.1	-	-	-	−92.6
**DABCO**	0.0	−26	−46.6	−0.9	−96.2	−107.6	−124.7	−92.6
**1,2-DMI**	0.0	−21.8	−33.5	7.2	−78.4	−86.9	−119.9	−92.6
**NEM**	0.0	−28.7	−49.1	−0.2	−95.9	−106	−132.2	−92.6
**Δ*H* (kJ/mol)**								
	**R**	**RC1**	**RC2**	**TS1**	**IM**	**TS2**	**PC**	**P**
**Cat.-free**	0.0	-	−8.9 *	116.5	-	-	-	−94.8
**DABCO**	0.0	−24.5	−41.4	−0.8	−96.9	−109.2	−124.6	−94.8
**1,2-DMI**	0.0	−20.6	−28.6	7.2	−78.8	−88.6	−119.6	−94.8
**NEM**	0.0	−27.3	−44.4	−0.9	−97.2	−107.9	−132.2	−94.8
**Δ*G* (kJ/mol)**								
	**R**	**RC1**	**RC2**	**TS1**	**IM**	**TS2**	**PC**	**P**
**Cat.-free**	0.0	-	28.9 *	170	-	-	-	−41.5
**DABCO**	0.0	14.5	28	91.5	2.2	−6.9	−28.8	−41.5
**1,2-DMI**	0.0	19.5	46.7	103.1	19.7	14.9	−24.6	−41.5
**NEM**	0.0	13.3	34.6	100.7	9.1	−0.9	−33.9	−41.5

* RC for catalyst-free (cat.-free) reaction.

**Table 3:** Computed (PA_calc_) and measured proton affinities (PA_exp_) of the tertiary amines of the studied catalysts: 1,4-diazabicyclo[2.2.2]octane (DABCO), 1,2-dimethylimidazole (1,2-DMI), and N-ethylmorpholine (NEM), in kJ/mol. The calculations were carried using the G3MP2BHandHLYP composite method in the gas phase at 298.15 K and 1 atm.

Catalysts	PA_calc_ (kJ/mol)	PA_exp_ (kJ/mol) [31]
**DABCO**	983.9	963.4
**1,2-DMI**	1002.9	984.7
**NEM**	973.2	-

**Table 4:** N-H, O-H, and C-O bond lengths (Å) along the phenyl isocyanate (PhNCO) and butan-1-ol reaction pathway in the presence of the studied catalysts: 1,4-diazabicyclo[2.2.2]octane (DABCO), 1,2-dimethylimidazole (1,2-DMI), and N-ethylmorpholine (NEM). N-H * for catalysts, while N-H ** for PhNCO.

Catalyst		RC1	RC2			TS1		IM		TS2			PC	
	N-H *	O-H	N-H *	O-H	C-O	N-H *	O-H	C-O	N-H *	N-H **	N-H *	N-H **	N-H *	N-H **
**DABCO**	1.856	0.976	1.835	0.979	3.073	1.661	1.012	1.853	1.074	1.668	1.207	1.367	1.998	1.023
**1,2-DMI**	1.878	0.973	1.898	0.972	3.059	1.704	1.001	1.832	1.082	1.610	1.150	1.450	1.972	1.020
**NEM**	1.926	0.974	1.920	0.976	2.958	1.744	1.006	1.808	1.070	1.713	1.214	1.388	2.067	1.020

## Data Availability

The data presented in this study are available on request from the corresponding author.

## Supplementary Materials

The following are available online at https://www.mdpi.com/article/10.3390/polym14142859/s1, Figure S1: HPLC chromatogram (246 nm) of the possible reaction products mixed at 100 ppm each; Figure S2: Optimized 3D structures of the catalyst-free system; Figure S3 and S4: Optimized structures of catalysts; Figure S5: Zero-point corrected relative energy (Δ*E*_0_, kJ/mol) of the reactant complex (RC1) *vs.* proton affinity (PA, kJ/mol) of the studied catalysts; Figure S6: Energy profile (zero-point corrected, Δ*E*_0_) of the catalyzed urethane formation reactions; Table S1: Relative entropies (Δ*S*), computed at the G3MP2BHandHLYP/6-31G(d) level of theory; Table S2: Zero-point corrected relative energies (Δ*E*_0_), relative enthalpies (Δ*H*), Gibbs free energies (Δ*G*), and entropies (Δ*S*) calculated at the BHandHLYP/6-31G(d) level of theory; Table S3: Cartesian coordinates of the optimized geometries.
