# Supplementary material for: Experimental and Theoretical Study of Cyclic Amine Catalysed Urethane Formation
Source: Polymers (Basel). 2022 Jul 13;14(14):2859. doi: 10.3390/polym14142859 (PMC9316557; doi:10.3390/polym14142859)
Supplement: Supplementary file 1 [file polymers-14-02859-s001.zip › polymers-1752518-supplementary.pdf]

Experimental and Theoretical Study of Cyclic Amine Catalysed Urethane Formation

Hadeer Q. Waleed[1], Dániel Pecsmány[1,2], Marcell Csécsi[1], László Farkas[3], Béla Viskolcz[1], Zsolt Fejes[1,*], Béla Fiser[1,2,4,*]

[1] Institute of Chemistry, University of Miskolc, 3515 Miskolc-Egyetemváros, Hungary
[2] Higher Education and Industrial Cooperation Centre, University of Miskolc, 3515 Miskolc-Egyetemváros, Hungary
[3] Wanhua-BorsodChem Zrt, Bolyai tér 1., H-3700 Kazincbarcika, Hungary
[4] Ferenc Rákóczi II, Transcarpathian Hungarian College of Higher Education, 90200 Beregszász, Transcarpathia, Ukraine
[*]Correspondence: kemfiser@uni-miskolc.hu (B.F.); kemfejes@uni-miskolc.hu (Zs.F.); Tel.: +36-46-565-111/1141 (B.F.); +36-46-565-111/1911 (Zs.F.)

Supporting Information

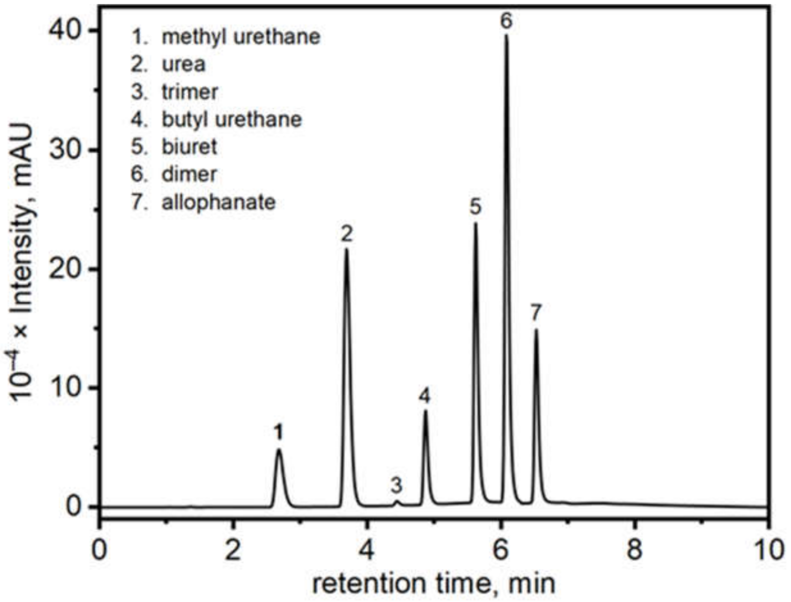

Figure S1. HPLC chromatogram (246 nm) of the possible reaction products mixed at 100 ppm each. Before the analysis, unreacted phenyl isocyanate (PhNCO) is converted to methyl urethane.

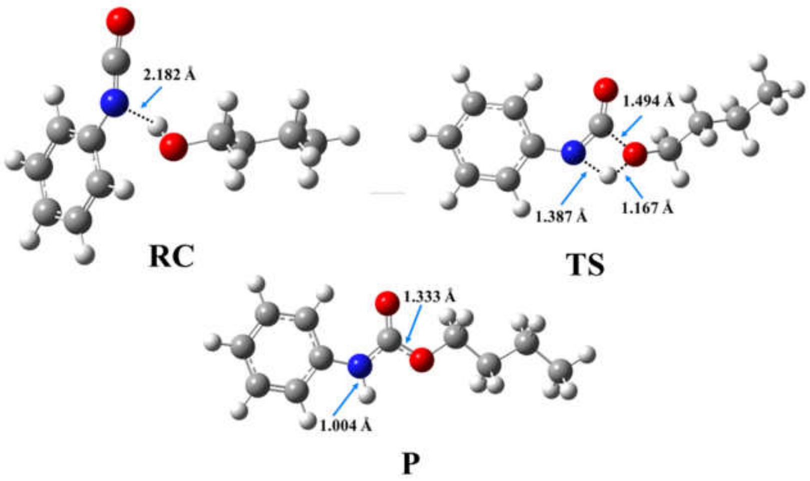

Figure S2. 3D structures of the reactant complex (RC), transition state (TS), and product (P) in the catalyst-free urethane formation. The structures have been optimized at the BHandHLYP/6-31G(d) level of theory in acetonitrile at 298.15 K and 1 atm.

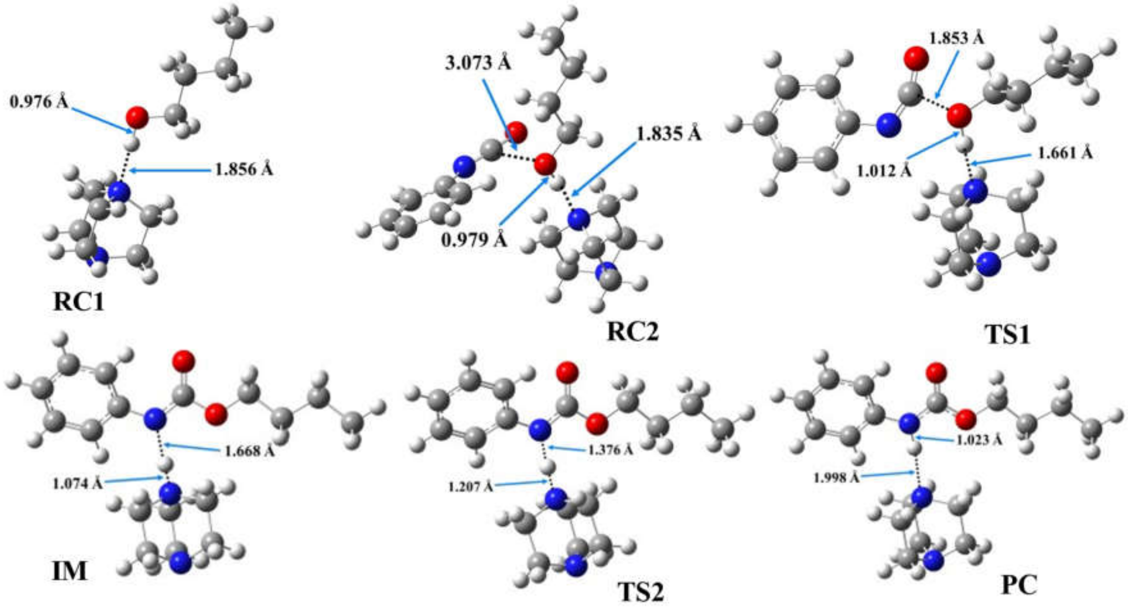

Figure S3. Optimized structures along the reaction pathway between phenyl isocyanate and butan-1-ol in the presence of 1,4-diazabicyclo[2.2.2]octane (DABCO) catalyst, calculated at the BHandHLYP/6-31G(d) level of theory in acetonitrile at 298.15 K and 1 atm. RC—reactant complex, TS—transition state, IM—intermediate, and PC—product complex.

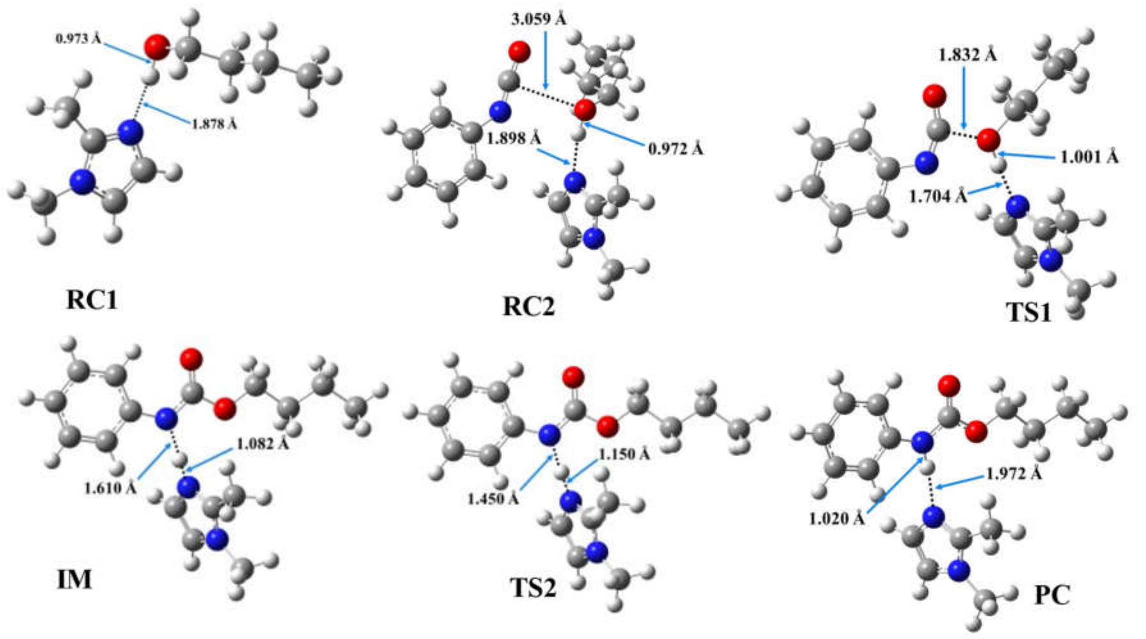

Figure S4. Optimized structures along the reaction pathway between phenyl isocyanate and butan-1-ol in the presence of 1,2-dimethylimidazole (1,2-DMI) catalyst, calculated at the BHandHLYP/6-31G(d) level of theory in acetonitrile at 298.15 K and 1 atm. RC—reactant complex, TS—transition state, IM—intermediate, and PC—product complex.

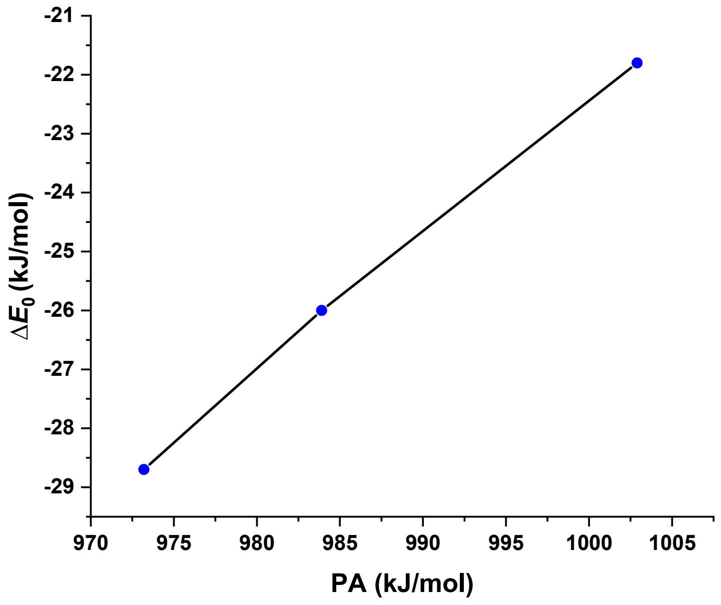

Figure S5. Zero-point corrected relative energy (ΔE_0, kJ/mol) of the reactant complex (RC1) vs. proton affinity (PA, kJ/mol) plot of the studied catalysts.

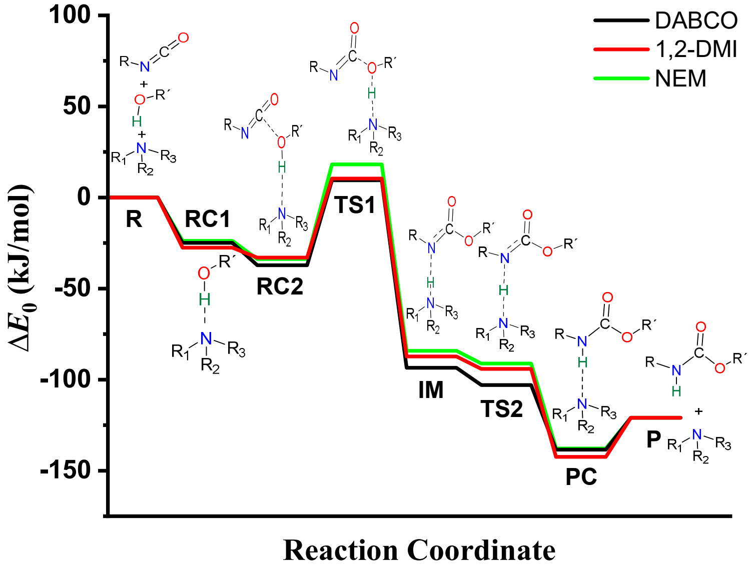

Figure S6. Energy profile (zero-point corrected, ΔE_0) of the studied catalyzed urethane formation reactions calculated at the BHandHLYP/6-31G(d) level of theory in acetonitrile using the SMD implicit solvent model at 298.15 K and 1 atm.

Table S1. Relative entropies (Δ*S*) of the reaction between phenyl isocyanate and butan-1-ol in presence of the studied catalysts, 1,4-diazabicyclo[2.2.2]octane (DABCO), 1,2-dimethylimidazole (1,2-DMI), and N-ethylmorpholine (NEM), calculated at the G3MP2BHandHLYP level of theory in acetonitrile using the SMD implicit solvent model at 298.15 K and 1 atm. R – reactant, RC – reactant complex, TS – transition state, IM – intermediate, PC – product complex, P – product.

	**Δ*S* (Cal/mol*K)**							
	**R**	**RC1**	**RC2**	**TS1**	**IM**	**TS2**	**PC**	**P**
**Cat.-free**	0.0	-	-30.4*	-97.3	-	-	-	-133.4
**DABCO**	0.0	-31.3	-55.7	-74.1	-79.4	-81.9	-76.9	-133.4
**1,2-DMI**	0.0	-32.1	-60.3	-76.8	-78.9	-82.9	-76.1	-133.4
**NEM**	0.0	-32.5	-63.4	-81.4	-85.2	-85.7	-78.8	-133.4

* RC for catalyst-free (cat.-free) reaction.

Table S2. Zero-point corrected relative energies (ΔE_0), enthalpies (Δ*H*), Gibbs free energies (Δ*G*), and entropies (Δ*S*) of the reaction between phenyl isocyanate and butan-1-ol in presence of the studied catalysts, 1,4-diazabicyclo[2.2.2]octane (DABCO), 1,2-dimethylimidazole (1,2-DMI), and N-ethylmorpholine (NEM), calculated at the BHandHLYP/6-31G(d) level of theory in acetonitrile using the SMD implicit solvent model at 298.15 K and 1 atm. R – reactant, RC – reactant complex, TS – transition state, IM – intermediate, PC – product complex, P – product.

	**ΔE_0 (kJ/mol)**							
	**R**	**RC1**	**RC2**	**TS1**	**IM**	**TS2**	**PC**	**P**
**Cat.-free**	0.0	-	-10.5*	122.8	-	-	-	-120.9
**DABCO**	0.0	-24.1	-37.2	9.5	-93.5	-102.9	-138.4	-120.9
**1,2-DMI**	0.0	-27.6	-32.1	10.4	-87.3	-94.2	-142.4	-120.9

**NEM**	0.0	-23.8	-33.8	18.2	-84.2	-91.2	-137.8	-120.9
				**ΔH (kJ/mol)**				
	**R**	**RC1**	**RC2**	**TS1**	**IM**	**TS2**	**PC**	**P**
**Cat.-free**	0.0	-	-8.2*	120.13	-	-	-	-123.2
**DABCO**	0.0	-23.5	-32.1	9.6	-94.2	-104.5	-138.3	-123.2
**1,2-DMI**	0.0	-26.3	-28.1	10.4	-87.6	-95.9	-142.1	-123.2
**NEM**	0.0	-22.4	-29.1	17.5	-85.5	-93.1	-137.8	-123.2
				**ΔG (kJ/mol)**				
	**R**	**RC1**	**RC2**	**TS1**	**IM**	**TS2**	**PC**	**P**
**Cat.-free**	0.0	-	29.7*	173.7	-	-	-	-69.9
**DABCO**	0.0	15.5	37.4	101.9	4.9	-2.2	-42.5	-69.9
**1,2-DMI**	0.0	13.8	47.2	106.2	10.9	7.6	-47.2	-69.9
**NEM**	0.0	18.2	49.9	119.1	20.8	13.9	-39.5	-69.9
				**ΔS (Cal/mol*Kelvin)**				
	**R**	**RC1**	**RC2**	**TS1**	**IM**	**TS2**	**PC**	**P**
**Cat.-free**	0.0	-	-30.4*	-97.3	-	-	-	-133.4
**DABCO**	0.0	-31.3	-55.7	-74.1	-79.5	-81.9	-76.9	-133.4
**1,2-DMI**	0.0	-32.1	-60.3	-76.8	-78.9	-82.9	-76.1	-133.4
**NEM**	0.0	-32.5	-63.4	-81.4	-85.3	-85.7	-78.8	-133.4

* RC for catalyst-free (cat.-free) reaction.

Table S3. Optimized geometries of the studied species. Optimization were carried out at the BHandHLYP/6-31G(d) level of theory in acetonitrile at 298.15 K and 1 atm. Cat.- catalysts: 1,4-diazabicyclo[2.2.2]octane (DABCO), 1,2-dimethylimidazole (1,2-DMI), and *N*-ethylmorpholine (NEM), RC – reactant complex, TS – transition state, IM – intermediate, PC – product complex.

**NEM**				**NEM-RC1**			
**N**	**-0.52505800**	**-0.23280900**	**0.17588200**	**O**	**0.63538400**	**0.60881600**	**1.78680500**
**C**	**-0.04657100**	**1.03464900**	**-0.35304800**	**N**	**-1.59582600**	**0.56966900**	**-0.04579300**
**C**	**0.41211800**	**-1.28762500**	**-0.17526700**	**C**	**-1.48119000**	**-0.03685400**	**-1.36833000**
**C**	**-1.87218000**	**-0.55709400**	**-0.26646400**	**C**	**-2.60231800**	**-0.14697800**	**0.73096900**
**H**	**-2.07595800**	**-1.57828900**	**0.04873200**	**C**	**-1.91094000**	**1.99472900**	**-0.10852200**
**H**	**-1.93852400**	**-0.54807000**	**-1.36261800**	**H**	**-2.13904500**	**2.31412900**	**0.90563600**
**C**	**1.79649000**	**-0.95074700**	**0.33785400**	**H**	**-2.81626100**	**2.15768300**	**-0.70413500**
**H**	**0.46016600**	**-1.43517500**	**-1.26247500**	**C**	**1.71109100**	**-0.24549200**	**1.48960700**
**H**	**0.08125700**	**-2.22307600**	**0.27054000**	**H**	**2.33138700**	**-0.28701000**	**2.38382200**
**C**	**1.34988700**	**1.31961500**	**0.16084300**	**H**	**-0.03616700**	**0.53331300**	**1.08525100**
**H**	**-0.70451300**	**1.83933500**	**-0.03898100**	**C**	**-2.26313300**	**-1.62053700**	**0.80160700**
**H**	**-0.03120800**	**1.02459800**	**-1.45136500**	**H**	**-3.59667000**	**-0.02605600**	**0.28633400**
**O**	**2.24439400**	**0.28368700**	**-0.17251800**	**H**	**-2.63251100**	**0.26614900**	**1.73604100**
**H**	**2.51275800**	**-1.70294900**	**0.02203100**	**C**	**-1.17903100**	**-1.51627900**	**-1.24702400**
**H**	**1.78527500**	**-0.91931100**	**1.43028200**	**H**	**-0.68150500**	**0.44180600**	**-1.92447500**
**H**	**1.32084800**	**1.45055200**	**1.24554800**	**H**	**-2.41130300**	**0.09686900**	**-1.93281000**
**H**	**1.73951400**	**2.22946400**	**-0.28521000**	**O**	**-2.15758200**	**-2.18333300**	**-0.48530400**
**C**	**-2.93653000**	**0.35491700**	**0.31784200**	**H**	**-3.04567800**	**-2.16301600**	**1.32260700**
**H**	**-2.85280600**	**1.37629400**	**-0.04396600**	**H**	**-1.32622400**	**-1.75659100**	**1.34714200**
**H**	**-2.87148000**	**0.37244700**	**1.40376300**	**H**	**-0.19464800**	**-1.65875600**	**-0.79414000**

H	-3.92435400	-0.00794000	0.04211500	H	-1.17299300	-1.97767000	-2.22953700
				H	1.36182700	-1.26478400	1.30237600
				C	-0.77389900	2.84450200	-0.64579400
				H	-0.54910900	2.62925900	-1.68696900
				H	0.12790500	2.68805800	-0.05933500
				H	-1.04350700	3.89635400	-0.58127600
				C	2.54804200	0.23064600	0.31426300
				H	1.91488800	0.28931800	-0.57293800
				H	2.89107000	1.24506200	0.52097200
				C	3.74145500	-0.67245500	0.02993800
				H	4.36787500	-0.73217700	0.92083700
				H	3.38924000	-1.68635100	-0.16409300
				C	4.58014800	-0.19545900	-1.14652100
				H	4.97510200	0.80351000	-0.96770100
				H	5.42452200	-0.85820600	-1.32599200
				H	3.98882900	-0.15738500	-2.06024100
**NEM-RC2**				**NEM-TS1**			
O	0.45648700	-0.71207800	-0.65890000	O	-0.62764100	0.56650000	-0.93729200
C	-1.46073100	-2.84534800	0.06455400	C	1.09146000	1.11650800	-0.83070500
N	0.85808500	1.78315700	0.73986700	C	-1.64025000	1.54865700	-1.14157200
C	2.22261400	2.17402000	1.08153500	H	-1.22455000	2.24870200	-1.85659500
C	0.27706600	2.78579900	-0.14799400	C	5.80523500	-0.11106200	0.28328800
C	0.01944100	1.59449000	1.92172400	C	5.25485600	1.07447300	-0.18071400
H	-1.00256400	1.47613000	1.57028200	C	4.97249300	-1.19689600	0.51511700
H	0.03848100	2.49426100	2.54697600	C	3.61095100	-1.09691300	0.28600000
C	1.61453600	-1.29526800	-1.20827500	C	3.05037100	0.09250700	-0.18024200
H	1.27648000	-2.10908200	-1.84527000	C	3.89234200	1.18263300	-0.41219400
C	-4.75776900	1.08190000	-0.69045300	H	3.48405000	2.11113600	-0.77261200
C	-3.53329300	0.88439500	-1.31425000	H	5.88874000	1.92735900	-0.36623400
C	-5.18138400	0.19190500	0.28518700	H	5.38301400	-2.12701100	0.87527100
C	-4.39186400	-0.89195400	0.63420200	H	2.96318400	-1.94025500	0.46238000
C	-3.17027300	-1.08621100	0.00134900	N	1.65924100	0.09596800	-0.38228400
C	-2.73293800	-0.19427500	-0.97416200	O	1.10122900	2.22870700	-1.23196900
H	-1.76894800	-0.34199600	-1.43083800	H	-0.90378500	-0.18384400	-0.32618700
H	-3.19594400	1.57322300	-2.07215700	H	-2.48764800	1.05496200	-1.60981900
H	-6.12940500	0.33758100	0.77724700	H	6.86568500	-0.18770600	0.46119000
H	-4.71280300	-1.59099900	1.38844600	O	-3.20805500	-2.86524200	-1.21315600
N	-2.41168200	-2.19394900	0.37042300	N	-1.35032000	-1.61670200	0.56258800
O	-0.58795700	-3.59584700	-0.12574700	C	-2.77756300	-1.72922900	0.87796400
H	0.69462600	0.11368000	-0.19598600	C	-0.96762800	-2.71156900	-0.33463700
C	1.14538900	2.97133900	-1.37385800	C	-3.61253700	-1.80222700	-0.38419700
H	0.17142700	3.74680400	0.36795800	C	-1.85045500	-2.73439300	-1.56379000
H	-0.71414900	2.45803400	-0.45058600	C	-0.52428500	-1.66100500	1.77398100
C	3.04286800	2.38675200	-0.17412700	C	-0.65739500	-0.43677000	2.65923300
H	2.68853100	1.39759200	1.67975100	H	-3.09123300	-0.86975500	1.46121900
H	2.21946800	3.09795900	1.67130100	H	-2.95442000	-2.62785500	1.47697900
O	2.45560100	3.34790200	-1.01957000	H	-1.04835900	-3.67135500	0.18423600
H	0.74649800	3.76112500	-2.00254600	H	0.06811300	-2.57281700	-0.63147500
H	1.16668600	2.04543400	-1.95385100	H	-4.65198800	-1.97723700	-0.12554000
H	3.15568000	1.44101400	-0.70961800	H	-3.55182700	-0.86002700	-0.93256700
H	4.03139700	2.75327700	0.08410500	H	-1.70102200	-1.82381200	-2.14828900
H	-5.37434900	1.92341700	-0.96141900	H	-1.59784500	-3.58605700	-2.18712400
H	2.13641300	-0.58196000	-1.85146300	H	-0.76288600	-2.56360100	2.34465300
C	0.39690400	0.37880000	2.74806500	H	0.50739100	-1.74819900	1.44881900
H	1.38653500	0.46564500	3.18860400	H	-0.38484200	0.46672700	2.12003900
H	0.36898800	-0.52092700	2.13874600	H	-1.66212300	-0.31290000	3.05387200
H	-0.31342600	0.25923300	3.56317900	H	0.01623900	-0.53521000	3.50756900

C	2.56904900	-1.83424000	-0.15740600	C	-2.05033200	2.26038400	0.13053300
H	2.87859300	-1.01803400	0.49784200	H	-2.43452600	1.53394600	0.84621600
H	2.02914000	-2.54928900	0.46272500	H	-1.16872400	2.71633300	0.57997500
C	3.80073500	-2.49570700	-0.76253600	C	-3.10636500	3.32741300	-0.13219400
H	4.32814300	-1.77497900	-1.38864700	H	-2.71969900	4.04607100	-0.85513400
H	3.48653300	-3.30333200	-1.42484400	H	-3.97957600	2.86600400	-0.59409900
C	4.75454400	-3.04567000	0.28785100	C	-3.52870900	4.05801700	1.13357900
H	4.26423200	-3.79331700	0.90955400	H	-4.27962200	4.81579100	0.91956300
H	5.62297600	-3.51433500	-0.17111600	H	-3.95152200	3.36852400	1.86261900
H	5.11352000	-2.25479700	0.94488500	H	-2.68005600	4.55434700	1.60159400
**NEM-IM**				**NEM-TS2**			
O	1.26280300	-0.97963200	0.07815100	O	-1.56442600	-0.80591800	0.02412100
C	0.03658000	-1.57639500	-0.04703700	C	-0.41127400	-1.52669700	-0.01870700
N	-0.16071400	1.96474100	0.52640900	C	-2.77086300	-1.54376900	-0.12394900
C	1.18593500	2.20835900	-0.05818800	H	-2.75924800	-2.07330400	-1.07289500
C	-1.17149600	2.79301300	-0.18762200	C	4.62046000	-2.10060100	0.02138300
C	-0.21380900	2.23111300	1.99206600	C	3.66116300	-2.67604800	-0.80055900
H	-1.24834300	2.08479100	2.28177500	C	4.23438300	-1.09226700	0.89186800
H	0.03552400	3.27762700	2.13573800	C	2.91445000	-0.67293200	0.94158400
C	2.39045600	-1.81122800	-0.14262500	C	1.93716300	-1.24249800	0.11763400
H	2.36713300	-2.65155200	0.54694400	C	2.34141500	-2.25661800	-0.76068200
H	2.35681800	-2.21692000	-1.15110300	H	1.61283700	-2.71604700	-1.40319900
C	-5.04930800	-1.53521700	-0.03598100	H	3.94219700	-3.45959100	-1.48759300
C	-4.14705100	-2.35022900	-0.70408600	H	4.96057700	-0.63180000	1.54382000
C	-4.55336400	-0.47299700	0.70830900	H	2.62371100	0.09886100	1.63582200
C	-3.19187800	-0.23809800	0.77861000	N	0.64156900	-0.72593700	0.17442400
C	-2.26094600	-1.05094200	0.10916000	O	-0.44702600	-2.73432800	-0.19328800
C	-2.78095300	-2.12206000	-0.63897500	H	0.42288900	0.63865300	0.30916100
H	-2.10373900	-2.77237600	-1.15894400	H	-2.84062200	-2.28699800	0.66611300
H	-4.50879200	-3.18130000	-1.29110000	H	5.64581100	-2.43185900	-0.01605100
H	-5.23026500	0.17646100	1.24245600	O	1.40475300	2.96433300	-1.90900700
H	-2.82080900	0.58409000	1.37030300	N	0.15773300	1.82015500	0.39938800
N	-0.92470700	-0.69242500	0.20755400	C	1.38717500	2.63974100	0.48510100
O	-0.02130300	-2.76555500	-0.33729700	C	-0.60262000	2.20066800	-0.81323700
H	-0.42354200	0.93581800	0.38896300	C	2.17944300	2.56714500	-0.80422900
C	-1.09166500	2.56036400	-1.68126500	C	0.27535100	2.13572600	-2.04420000
H	-0.96354300	3.83205300	0.04763900	C	-0.70160400	1.97636700	1.59791200
H	-2.15045500	2.52673100	0.19198100	C	-0.06515100	1.47161800	2.87710600
C	1.15195900	2.00552500	-1.55835200	H	1.99338500	2.28484600	1.30863700
H	1.88509300	1.52018400	0.39321100	H	1.09438800	3.66901800	0.68392500
H	1.46283800	3.22937700	0.18653400	H	-0.97931800	3.21234100	-0.67798700
O	0.19694800	2.83815600	-2.17057900	H	-1.43790900	1.51799800	-0.90888600
H	-1.78195300	3.23082800	-2.18136400	H	3.02359000	3.24570500	-0.74113100
H	-1.37318800	1.53379200	-1.91998400	H	2.56230300	1.55739100	-0.95775400
H	0.94534600	0.96006000	-1.78731100	H	0.58445000	1.10658300	-2.23489000
H	2.12043300	2.26522300	-1.97177800	H	-0.28397700	2.48938300	-2.90375500
H	-6.10961700	-1.72309900	-0.09183000	H	-0.96577400	3.02817600	1.68557000
C	0.69183700	1.32756300	2.80121700	H	-1.60620300	1.41616300	1.39272700
H	1.74346400	1.53510600	2.63034100	H	0.22048200	0.42744700	2.79009000
H	0.48884000	1.49839200	3.85512100	H	0.80743800	2.04984500	3.16615500
H	0.50100400	0.28169100	2.58166700	H	-0.79330200	1.55463800	3.68014900
C	3.63925300	-0.98295800	0.06304700	C	-3.93008400	-0.57550300	-0.05802200
H	3.62466300	-0.13599900	-0.62319600	H	-3.82223500	0.16748900	-0.84822500
H	3.63298600	-0.57336800	1.07323100	H	-3.89508400	-0.03907800	0.89011200
C	4.90988500	-1.79538400	-0.15334600	C	-5.27245700	-1.28270900	-0.19830900
H	4.90919400	-2.20422000	-1.16410700	H	-5.37151200	-2.02852900	0.59076000
H	4.91171400	-2.65025200	0.52350200	H	-5.29618700	-1.82873400	-1.14172200

**C**	**6.17417300**	**-0.97706900**	**0.06154500**	**C**	**-6.45048600**	**-0.32205400**	**-0.13895800**
**H**	**6.21880500**	**-0.58256700**	**1.07556300**	**H**	**-7.39625200**	**-0.85060300**	**-0.23954200**
**H**	**7.06637300**	**-1.57885700**	**-0.09925500**	**H**	**-6.47063400**	**0.21683700**	**0.80708000**
**H**	**6.21672100**	**-0.13183000**	**-0.62380700**	**H**	**-6.39613000**	**0.41552900**	**-0.93818600**
**NEM-PC**							
**O**	**1.27832700**	**-1.10720300**	**0.01691200**				
**C**	**0.10316100**	**-1.73382800**	**-0.09799400**				
**N**	**-0.20393900**	**2.11481200**	**0.54616800**				
**C**	**1.14607600**	**2.33365600**	**0.03506700**				
**C**	**-1.13143100**	**2.98563700**	**-0.16944000**				
**C**	**-0.30077000**	**2.34860800**	**1.98402800**				
**C**	**0.48103600**	**1.35660200**	**2.82613000**				
**H**	**-1.35434700**	**2.28078900**	**2.24598200**				
**H**	**0.01971700**	**3.36927800**	**2.22491200**				
**H**	**1.55458600**	**1.44887900**	**2.68579900**				
**H**	**0.19415900**	**0.33379900**	**2.59418100**				
**C**	**2.44563600**	**-1.92549300**	**-0.10602700**				
**H**	**2.42592000**	**-2.68709800**	**0.66780200**				
**C**	**-5.04569000**	**-1.55924500**	**-0.01465200**				
**C**	**-4.18240600**	**-2.49411800**	**-0.56414400**				
**C**	**-4.51720900**	**-0.40908000**	**0.55339500**				
**C**	**-3.14923200**	**-0.20181000**	**0.57484300**				
**C**	**-2.28067500**	**-1.14119800**	**0.01809700**				
**C**	**-2.80985400**	**-2.29516300**	**-0.55769900**				
**H**	**-2.15403900**	**-3.02663100**	**-0.98882000**				
**H**	**-4.57607400**	**-3.39277900**	**-1.01228700**				
**H**	**-5.16963800**	**0.33077400**	**0.98921700**				
**H**	**-2.74450100**	**0.68731800**	**1.03013000**				
**N**	**-0.91061300**	**-0.84678800**	**0.04509300**				
**O**	**-0.00046800**	**-2.92246700**	**-0.29875100**				
**H**	**-0.64982500**	**0.11879800**	**0.24549200**				
**C**	**-1.03754700**	**2.75567000**	**-1.66291900**				
**H**	**-0.91449800**	**4.03920300**	**0.04307500**				
**H**	**-2.14450400**	**2.78062700**	**0.16639100**				
**C**	**1.18316700**	**2.12175000**	**-1.46435400**				
**H**	**1.83141500**	**1.63685900**	**0.50379900**				
**H**	**1.48376500**	**3.35152600**	**0.26500600**				
**O**	**0.27391100**	**2.96740900**	**-2.13032400**				
**H**	**-1.67997900**	**3.45365400**	**-2.19073500**				
**H**	**-1.36006400**	**1.74024800**	**-1.90542300**				
**H**	**0.95782800**	**1.07880100**	**-1.69451500**				
**H**	**2.17124900**	**2.35363800**	**-1.84985600**				
**H**	**-6.11085200**	**-1.72366500**	**-0.02764800**				
**H**	**2.42928400**	**-2.42321800**	**-1.07081300**				
**H**	**0.27403500**	**1.53515600**	**3.87898800**				
**C**	**3.65501300**	**-1.03158000**	**0.03216900**				
**H**	**3.61215900**	**-0.51898600**	**0.99260000**				
**H**	**3.62245300**	**-0.26559700**	**-0.74203200**				
**C**	**4.95552100**	**-1.81851900**	**-0.07463700**				
**H**	**4.98262100**	**-2.34417300**	**-1.02929600**				
**H**	**4.97825600**	**-2.58510200**	**0.70005400**				
**C**	**6.18537000**	**-0.93229000**	**0.05189200**				
**H**	**6.19974000**	**-0.41501900**	**1.00993800**				
**H**	**7.10062600**	**-1.51567600**	**-0.02383900**				
**H**	**6.20722000**	**-0.17708000**	**-0.73215400**				
**DABCO**				**DABCO-RC1**			
**C**	**-1.18978300**	**-0.77507000**	**-0.68701100**	**O**	**-1.43993500**	**-1.09066800**	**0.02895800**

**H**	**-1.18952200**	**-1.16889200**	**-1.70067800**	**C**	**-2.29502600**	**0.02038600**	**0.06925900**
**H**	**-2.06783100**	**-1.16936300**	**-0.18083700**	**H**	**-2.08995900**	**0.70723000**	**-0.75827200**
**N**	**0.00002300**	**1.27717600**	**0.00020800**	**H**	**-0.51810400**	**-0.76781600**	**0.04152100**
**N**	**-0.00002300**	**-1.27717600**	**0.00020800**	**H**	**-2.15656400**	**0.59262800**	**0.99269900**
**C**	**1.18978300**	**0.77507000**	**-0.68701100**	**C**	**2.94012400**	**1.54079000**	**-0.39095900**
**H**	**1.18952200**	**1.16889200**	**-1.70067800**	**H**	**3.18272700**	**2.33987800**	**0.30502300**
**H**	**2.06783100**	**1.16936300**	**-0.18083700**	**H**	**3.23701500**	**1.86740800**	**-1.38438200**
**C**	**1.18976400**	**-0.77511300**	**-0.68699500**	**N**	**1.25269600**	**-0.20947100**	**0.02348500**
**H**	**2.06779000**	**-1.16941700**	**-0.18079200**	**N**	**3.73461300**	**0.36542300**	**-0.03755800**
**H**	**1.18951800**	**-1.16895500**	**-1.70065500**	**C**	**1.85065200**	**-0.43912500**	**1.34228400**
**C**	**-1.18976400**	**0.77511300**	**-0.68699500**	**H**	**1.31326900**	**0.16585700**	**2.06775000**
**H**	**-1.18951800**	**1.16895500**	**-1.70065500**	**H**	**1.69863200**	**-1.48294100**	**1.60382200**
**H**	**-2.06779000**	**1.16941700**	**-0.18079200**	**C**	**3.35608200**	**-0.07629600**	**1.30416200**
**C**	**0.00002300**	**0.77480800**	**1.37390900**	**H**	**3.97230600**	**-0.93152400**	**1.56988600**
**H**	**-0.87746300**	**1.16915000**	**1.88105500**	**H**	**3.58370600**	**0.72457600**	**2.00304000**
**H**	**0.87754500**	**1.16909700**	**1.88103400**	**C**	**1.42968600**	**1.19924800**	**-0.34382400**
**C**	**-0.00002300**	**-0.77480800**	**1.37390900**	**H**	**0.90682900**	**1.80756900**	**0.38929600**
**H**	**0.87746300**	**-1.16915000**	**1.88105500**	**H**	**0.95518800**	**1.36045400**	**-1.30818500**
**H**	**-0.87754500**	**-1.16909700**	**1.88103400**	**C**	**1.93599100**	**-1.05082900**	**-0.96442400**
				**H**	**1.47138200**	**-0.87951800**	**-1.93180400**
				**H**	**1.76861000**	**-2.09071100**	**-0.69645600**
				**C**	**3.44580900**	**-0.70659300**	**-0.98935400**
				**H**	**4.04923900**	**-1.57065100**	**-0.72301900**
				**H**	**3.75710300**	**-0.37961600**	**-1.97826900**
				**C**	**-3.73075100**	**-0.45349500**	**-0.01830000**
				**H**	**-3.91521800**	**-1.15455200**	**0.79643900**
				**H**	**-3.85499100**	**-1.01298200**	**-0.94614400**
				**C**	**-4.74226200**	**0.68328200**	**0.03961100**
				**H**	**-4.53866700**	**1.39027000**	**-0.76547700**
				**H**	**-4.61264900**	**1.23498600**	**0.97136200**
				**C**	**-6.18081200**	**0.19909300**	**-0.06556900**
				**H**	**-6.42523300**	**-0.48453200**	**0.74615700**
				**H**	**-6.88415800**	**1.02843100**	**-0.02276900**
				**H**	**-6.34910600**	**-0.32952300**	**-1.00269900**
**DABCO-RC2**				**DABCO-TS1**			
**O**	**0.46240500**	**1.17818600**	**-0.60773200**	**O**	**-0.66483400**	**1.26577500**	**-0.08517000**
**C**	**1.21784800**	**-0.24919900**	**2.00764800**	**C**	**1.10675700**	**1.68121000**	**0.26809800**
**C**	**0.54038900**	**2.55307400**	**-0.32234900**	**C**	**-1.68506400**	**2.02864600**	**0.54578300**
**H**	**0.19694000**	**2.75733400**	**0.69382900**	**H**	**-1.19642100**	**2.88765300**	**0.99413700**
**C**	**3.13422800**	**-3.66133600**	**-1.43303000**	**C**	**5.85276500**	**0.19186600**	**-0.18658600**
**C**	**2.61935200**	**-2.40378500**	**-1.71567000**	**C**	**5.30517900**	**1.34462300**	**0.35667600**
**C**	**3.16099200**	**-4.11287100**	**-0.12165000**	**C**	**5.00781300**	**-0.77252900**	**-0.71781100**
**C**	**2.67794200**	**-3.31493800**	**0.90240500**	**C**	**3.63618100**	**-0.58573500**	**-0.70501600**
**C**	**2.16499100**	**-2.05695800**	**0.60988000**	**C**	**3.07944700**	**0.57085700**	**-0.15992900**
**C**	**2.13289700**	**-1.59373200**	**-0.70238000**	**C**	**3.93281900**	**1.53901000**	**0.37316800**
**H**	**1.72611200**	**-0.61710500**	**-0.90959000**	**H**	**3.52613300**	**2.44029100**	**0.79931700**
**H**	**2.59481500**	**-2.04726100**	**-2.73301900**	**H**	**5.94883100**	**2.10342900**	**0.77319100**
**H**	**3.55851100**	**-5.08818400**	**0.10820200**	**H**	**5.41662000**	**-1.67462800**	**-1.14486400**
**H**	**2.69360800**	**-3.65468300**	**1.92455200**	**H**	**2.97741800**	**-1.33213800**	**-1.11844600**
**N**	**1.68917600**	**-1.28585600**	**1.66506000**	**N**	**1.67681200**	**0.67109100**	**-0.19242800**
**O**	**0.76285400**	**0.70791700**	**2.49992700**	**O**	**1.08696900**	**2.74299800**	**0.78202600**
**H**	**-0.45980700**	**0.88001100**	**-0.46478400**	**H**	**-0.85902000**	**0.27379800**	**-0.02603000**
**H**	**-0.10123000**	**3.12397900**	**-1.00030100**	**H**	**-2.12863000**	**1.44023300**	**1.34679600**
**H**	**3.51059900**	**-4.28404400**	**-2.22826700**	**H**	**6.92094500**	**0.04723000**	**-0.19602500**
**C**	**-4.44591800**	**0.58168400**	**0.66484200**	**C**	**-3.04360000**	**-3.03676500**	**0.28220500**
**H**	**-5.18434400**	**1.25621500**	**0.23891700**	**H**	**-3.72552900**	**-3.29389700**	**-0.52399900**
**H**	**-4.71380800**	**0.41269000**	**1.70473900**	**H**	**-3.51344400**	**-3.32730800**	**1.21811800**

N	-2.17549800	0.26693300	-0.24404500	N	-1.27494600	-1.33145100	0.06978300
N	-4.52663900	-0.69488900	-0.04385100	N	-1.82068900	-3.81911100	0.11708300
C	-2.76427100	0.10514400	-1.57734900	C	-0.88551200	-1.95423300	-1.20391500
H	-2.77249000	1.07749900	-2.06262100	H	-1.43077700	-1.45408500	-1.99935800
H	-2.11631800	-0.54848300	-2.15518100	H	0.17287600	-1.76866900	-1.35830000
C	-4.19301500	-0.47850900	-1.45090300	C	-1.20149100	-3.46840900	-1.15944600
H	-4.27497600	-1.42915300	-1.97173300	H	-0.29602100	-4.05862000	-1.27286000
H	-4.93092800	0.19633100	-1.87725500	H	-1.88144200	-3.74827200	-1.95973400
C	-3.01823100	1.17085700	0.54553500	C	-2.71846800	-1.52289300	0.27270600
H	-3.02745300	2.14024400	0.05441600	H	-3.24259900	-1.00628700	-0.52626300
H	-2.55592400	1.29676300	1.52075200	H	-2.99129800	-1.05108700	1.21211800
C	-2.12515200	-1.04025800	0.41950900	C	-0.54632000	-1.98158900	1.16832100
H	-1.67004900	-0.90496000	1.39693700	H	-0.82398800	-1.48657400	2.09459800
H	-1.47702700	-1.69009600	-0.16240100	H	0.51475900	-1.81850900	1.00803000
C	-3.55612700	-1.62177100	0.53659900	C	-0.89595000	-3.48924600	1.19999100
H	-3.63623000	-2.57175400	0.01437100	H	-0.00296400	-4.09796600	1.08533200
H	-3.82541600	-1.79210000	1.57591000	H	-1.36328700	-3.76142300	2.14269400
C	1.97383900	3.01540100	-0.47458600	C	-2.73330300	2.47279600	-0.45390100
H	2.30998400	2.78380500	-1.48588100	H	-3.17811500	1.59578100	-0.92471400
H	2.59995800	2.43547300	0.20446100	H	-2.24162400	3.04057100	-1.24370300
C	2.15296500	4.50219400	-0.19794700	C	-3.82325900	3.31864800	0.19202300
H	1.51871400	5.07515200	-0.87531900	H	-4.30196600	2.74597000	0.98698900
H	1.80410300	4.72624700	0.81070300	H	-3.36922600	4.18698600	0.67039500
C	3.59588600	4.96138100	-0.34725500	C	-4.87659200	3.78101100	-0.80360400
H	3.96301600	4.78008000	-1.35635100	H	-5.37117900	2.93286200	-1.27455000
H	3.69634200	6.02590100	-0.14459900	H	-5.64194500	4.38291100	-0.31779000
H	4.25026100	4.42969800	0.34200900	H	-4.43184200	4.38426200	-1.59355600
	**DABCO-IM**				**DABCO-TS2**		
O	1.23762000	-1.34096200	0.08207200	O	-1.12758700	-1.31431600	-0.10470600
C	-0.00222400	-1.91525300	0.04176600	C	0.13325600	-1.82437400	-0.07728000
C	2.34127000	-2.22852300	0.01583100	C	-2.18761600	-2.25977900	-0.04502000
H	2.29267700	-2.93693400	0.83957400	H	-2.10562700	-2.95064900	-0.88007900
H	2.29918500	-2.80140200	-0.90763400	H	-2.10628500	-2.84013500	0.87041000
C	-5.08424900	-1.72325400	-0.00135900	C	5.19197700	-1.45153100	0.02971800
C	-4.20555400	-2.64514200	-0.55198000	C	4.33969300	-2.30059400	0.72195900
C	-4.55874600	-0.58746400	0.60031800	C	4.64184300	-0.42942100	-0.72993200
C	-3.19093000	-0.38513100	0.64668200	C	3.26814100	-0.26440700	-0.79669600
C	-2.28438300	-1.30494100	0.09308100	C	2.39678300	-1.10972300	-0.10020600
C	-2.83353700	-2.44965100	-0.51039600	C	2.96496300	-2.13699800	0.66511100
H	-2.17419000	-3.18186200	-0.93634500	H	2.32195500	-2.80609700	1.20634800
H	-4.59119100	-3.53516400	-1.02653200	H	4.74804800	-3.10037800	1.32082800
H	-5.21728800	0.14540000	1.04138300	H	5.28279100	0.24079200	-1.28173300
H	-2.79511200	0.49591200	1.12729500	H	2.85086100	0.52154800	-1.40542600
N	-0.93854800	-0.97594200	0.14762400	N	1.03038500	-0.83797200	-0.16601700
O	-0.09681200	-3.13312600	-0.05819500	O	0.30787100	-3.03026200	-0.00154400
H	-0.42598500	0.60883900	0.04870300	H	0.57795800	0.46173600	-0.12402200
H	-6.14954200	-1.88605800	-0.03777500	H	6.26065900	-1.58537900	0.07988000
C	1.53223900	3.28474200	-0.70454800	C	-1.25885900	3.06432400	1.35083500
H	1.69713600	3.58825800	-1.73332300	H	-0.96292300	3.56126100	2.26970500
H	2.43711100	3.49450200	-0.14331500	H	-2.34163100	2.98292800	1.34935100
N	-0.15372700	1.64591700	-0.01869200	N	0.13767600	1.58200600	-0.02228200
N	0.44591900	4.08682600	-0.15224100	N	-0.85871500	3.89620400	0.21953400
C	-1.16268900	2.34901700	-0.85530500	C	1.22350000	2.58791600	-0.02588100
H	-1.15829100	1.87259200	-1.82847100	H	1.92197300	2.32509900	0.76056700
H	-2.12951100	2.19171000	-0.39443000	H	1.73451900	2.51364000	-0.97882200
C	-0.77079300	3.84087800	-0.91834500	C	0.59764000	3.98542700	0.18672800
H	-1.56636200	4.45779800	-0.51330700	H	0.88808800	4.65731400	-0.61519400

H	-0.60004800	4.14451100	-1.94609200	H	0.93534300	4.41780300	1.12368400
C	1.19336800	1.78008900	-0.63368100	C	-0.60697100	1.66762000	1.25434100
H	1.14782000	1.31382000	-1.61032200	H	0.10418600	1.48963200	2.05387400
H	1.88248800	1.22091700	-0.01627500	H	-1.33499600	0.86629100	1.25862000
C	-0.14127700	2.21671800	1.35333200	C	-0.77746400	1.84340000	-1.15492900
H	0.57985400	1.64880100	1.92899200	H	-1.55773500	1.09352000	-1.11695900
H	-1.12619600	2.06074100	1.77699400	H	-0.21175400	1.70370000	-2.06993400
C	0.22709100	3.71206700	1.24079300	C	-1.32829400	3.28035900	-1.01879600
H	-0.56947000	4.32896200	1.64395800	H	-1.00341900	3.89694300	-1.85138900
H	1.13176400	3.92224300	1.80200200	H	-2.41405700	3.27256100	-1.01300100
C	3.61295900	-1.41252700	0.08463100	C	-3.49761700	-1.50660600	-0.09388100
H	3.63048800	-0.70626300	-0.74530100	H	-3.53566900	-0.91420500	-1.00801100
H	3.61012700	-0.82421000	1.00236400	H	-3.54049500	-0.80717100	0.74100300
C	4.85894300	-2.28845700	0.03999900	C	-4.69738700	-2.44440900	-0.03940900
H	4.83319500	-2.99469900	0.87024300	H	-4.64421800	-3.14718500	-0.87123100
H	4.84766000	-2.88627900	-0.87180600	H	-4.64762000	-3.04190500	0.87122600
C	6.14724800	-1.48170300	0.10099800	C	-6.02459100	-1.70248100	-0.08680900
H	7.02103200	-2.12945400	0.06951400	H	-6.86443200	-2.39312300	-0.04622300
H	6.21719300	-0.78960000	-0.73681900	H	-6.12013300	-1.01451800	0.75185800
H	6.20107100	-0.89676700	1.01795600	H	-6.11707600	-1.12138900	-1.00307500

DABCO-PC

O	1.19187600	-1.50370400	0.02966500
C	-0.01629000	-2.07383000	-0.00430700
C	2.30968600	-2.39593300	0.01080300
H	2.24370900	-3.06426900	0.86415600
H	2.26745000	-2.99740500	-0.89239300
C	-5.14834700	-1.60888600	0.05097400
C	-4.33826000	-2.67162100	-0.31636100
C	-4.55557900	-0.40642000	0.40825300
C	-3.17845000	-0.27315800	0.40199000
C	-2.36216700	-1.34191400	0.02866800
C	-2.95671000	-2.54984600	-0.33516700
H	-2.34360200	-3.38150700	-0.62309600
H	-4.78104800	-3.61361400	-0.59950200
H	-5.16497700	0.43417700	0.70001300
H	-2.72641600	0.66080000	0.69236100
N	-0.97881400	-1.12166000	0.01931500
O	-0.18411400	-3.27199100	-0.04691700
H	-0.66362300	-0.14992800	0.07611000
H	-6.22094600	-1.71492200	0.05971200
C	1.72748100	3.33200400	-0.43969800
H	2.08319800	3.57360400	-1.43829800
H	2.53449800	3.54311900	0.25765200
N	-0.13504400	1.77628200	0.01494800
N	0.60614000	4.21650000	-0.12590000
C	-0.91971200	2.48257500	-1.00302400
H	-0.77260800	1.97513000	-1.95293900
H	-1.97102800	2.39731700	-0.74282000
C	-0.47466900	3.96430400	-1.07764400
H	-1.29914500	4.63287000	-0.84243100
H	-0.11946700	4.21713200	-2.07359800
C	1.28425000	1.85069500	-0.34724300
H	1.41850400	1.33490500	-1.29390300
H	1.85180100	1.31093000	0.40435600
C	-0.32807300	2.43620100	1.30973100
H	0.24021800	1.88797500	2.05660100
H	-1.37949400	2.36341000	1.57516300

C	0.12939800	3.91379900	1.22278200
H	-0.68802100	4.59132100	1.45678800
H	0.93614100	4.11568700	1.92299500
C	3.57026400	-1.56565000	0.05977200
H	3.55907600	-0.95160800	0.95968000
H	3.58433500	-0.88624700	-0.79161000
C	4.82066900	-2.43664200	0.04546600
H	4.81818800	-3.05741600	-0.85068500
H	4.79433300	-3.12052000	0.89399000
C	6.10206500	-1.61823500	0.09271600
H	6.14634900	-1.01028800	0.99496600
H	6.98027900	-2.26043600	0.08195000
H	6.17090200	-0.94717800	-0.76200100

1,2-DMI				1,2-DMI-RC1			
C	0.24815900	-0.68189500	0.00000000	N	-1.00004100	-0.17881400	-0.60215400
C	-1.84344500	-0.24030300	0.00000000	H	0.46681800	0.93957500	-0.95606600
C	-1.20620500	0.95897000	0.00000000	C	-1.34047400	-1.49344900	-0.78444300
N	0.13292300	0.66746100	0.00000100	C	2.09112500	1.31687600	0.02570800
H	-2.90098400	-0.42909200	-0.00000100	H	1.58538100	1.15484200	0.98181800
H	-1.56132700	1.97062600	0.00000200	H	2.72309700	2.19539300	0.14824800
N	-0.92896700	-1.26082400	0.00000000	C	-2.58809300	-1.71486900	-0.29961500
C	1.21824200	1.62415300	-0.00000100	N	-3.01909900	-0.50897500	0.19135200
H	1.83627700	1.50160300	-0.88400400	C	-2.02998200	0.38704800	-0.01463800
H	1.83629500	1.50158400	0.88398700	H	-3.20184200	-2.59260000	-0.25326400
H	0.80013900	2.62280000	0.00001400	H	-0.67024300	-2.19230800	-1.24809100
C	1.56084700	-1.38011200	0.00000000	C	-4.30738100	-0.24100400	0.79459700
H	2.15177100	-1.12377500	-0.87758300	H	-4.17888500	0.23789400	1.75941500
H	2.15177200	-1.12377500	0.87758300	H	-4.82589600	-1.18080300	0.93491300
H	1.39277200	-2.45131300	0.00000000	H	-4.90498700	0.40128400	0.15495300
				O	1.16320700	1.61906700	-0.98577700
				C	2.95306500	0.11023900	-0.30577600
				H	2.30630700	-0.75921500	-0.43714600
				H	3.44012300	0.28544200	-1.26571800
				C	3.99935500	-0.18695800	0.76038500
				H	3.50335000	-0.34972800	1.71806100
				H	4.63751500	0.68778500	0.89199200
				C	4.86159500	-1.39536000	0.42633400
				H	5.39813500	-1.24791000	-0.50981300
				H	5.59896000	-1.58408800	1.20429400
				H	4.25464100	-2.29332500	0.32032300
				C	-2.12059000	1.81742500	0.37841200
				H	-2.95347500	2.31471700	-0.11488000
				H	-2.26153300	1.92866500	1.45183100
				H	-1.20086400	2.31668400	0.09517400

1,2-DMI-RC2				1,2-DMI-TS1			
C	-1.30679900	2.48941800	0.64832600	C	1.40612400	1.19574400	-0.83159900
N	1.16531600	-1.39644400	-0.17888100	N	-1.68075000	-0.99704500	-0.08116500
H	1.12906300	0.24764400	0.76885700	H	-0.82872900	0.30219300	-0.78271600
C	1.10971500	-1.81813300	-1.48228400	C	-1.30274700	-1.62358500	1.07790500
C	2.52856700	1.53974000	1.12749400	C	-1.15646800	2.27644000	-1.06123100
H	3.29957500	0.76500200	1.16888200	H	-2.09456000	2.12703100	-1.59145900
H	2.73728900	2.23658900	1.93772100	H	-0.60394700	3.05682600	-1.57235400
C	-5.29915600	-0.53727200	-0.57601000	C	5.48056100	-1.34281800	0.56703100
C	-5.32280200	0.81119700	-0.24706000	C	5.31708200	-0.01841900	0.18847500
C	-4.08974300	-1.21596200	-0.60258400	C	4.37626300	-2.18368800	0.58041200
C	-2.90899300	-0.55573900	-0.30369800	C	3.12811000	-1.70560200	0.22027100
C	-2.93956900	0.79359700	0.02423900	C	2.95690800	-0.37455000	-0.15999500

C	-4.14921500	1.48146000	0.05348500	C	4.07030200	0.46805400	-0.17296600
H	-6.25818200	1.34666600	-0.22361100	H	6.16607300	0.64688600	0.17305600
H	-4.06189200	-2.26325600	-0.85641500	H	4.48548800	-3.21638700	0.87192200
H	-1.96311900	-1.07086700	-0.32020800	H	2.26676800	-2.35376800	0.22825100
N	-1.73283200	1.42403100	0.31867300	N	1.65236600	0.01526700	-0.50652600
O	-0.80408500	3.49455600	0.95778100	O	1.70705000	2.32000500	-1.03380600
C	1.34619200	-3.15277200	-1.54217600	C	-2.16105600	-2.63872500	1.34292400
N	1.55156600	-3.55575400	-0.24749500	N	-3.07997300	-2.63144400	0.32438200
C	1.43306900	-2.46448600	0.53872900	C	-2.75206500	-1.62539000	-0.51200400
H	1.38910500	-3.84461600	-2.35995900	H	-2.21074000	-3.35446900	2.13926500
H	0.90538800	-1.13869200	-2.28790000	H	-0.44359600	-1.30518200	1.63616800
H	-4.16235200	2.52837200	0.30975700	H	3.96203800	1.49914400	-0.46368200
C	1.83831700	-4.90412200	0.19452300	C	-4.19817800	-3.53988700	0.17512300
H	1.06573500	-5.25542500	0.87096600	H	-4.11793700	-4.09044700	-0.75648400
H	2.79811300	-4.94293600	0.69959600	H	-5.13577700	-2.99363200	0.18751700
H	1.86932300	-5.55209500	-0.67200100	H	-4.18559800	-4.23927900	1.00115800
O	1.25883200	0.99205500	1.38141600	O	-0.38327700	1.09012700	-1.21178300
H	-6.21605100	-1.05368600	-0.80903100	H	6.45310800	-1.71382800	0.84738200
C	2.60678600	2.26569600	-0.20486500	C	-1.40083200	2.64690500	0.38761800
H	2.37824300	1.55941900	-1.00513400	H	-1.92322600	1.83022400	0.88517700
H	1.82935400	3.02941300	-0.22845300	H	-0.44081200	2.76464800	0.88959700
C	3.96827300	2.90003000	-0.45768400	C	-2.21087600	3.93082400	0.51818400
H	4.19344500	3.60186500	0.34636800	H	-1.68538000	4.74102000	0.01199800
H	4.73947100	2.12984300	-0.41546700	H	-3.16308000	3.81143100	0.00048000
C	4.04815100	3.62156400	-1.79494600	C	-2.46706400	4.31887400	1.96662400
H	3.31037800	4.42039100	-1.85471900	H	-1.53216400	4.47773200	2.50173200
H	5.02994500	4.06550500	-1.94840400	H	-3.04692400	5.23728900	2.03212800
H	3.86097200	2.93779300	-2.62164800	H	-3.01908000	3.53962200	2.48981000
C	1.58940500	-2.49558700	2.01629500	C	-3.51815200	-1.30199200	-1.74264000
H	0.83856700	-3.12991400	2.48372500	H	-3.56905100	-2.15751100	-2.41284800
H	2.56672900	-2.87672700	2.30567500	H	-4.53896900	-1.00633300	-1.50864600
H	1.48139300	-1.48778600	2.40103500	H	-3.03306900	-0.48480500	-2.26437000
**1,2-DMI-IM**				**1,2-DMI-TS2**			
C	4.68458600	0.76096000	0.06769400	C	4.66127400	0.62223100	0.08643100
C	5.47869100	-0.37905400	0.09100000	C	5.42007400	-0.54122200	0.09948400
C	4.85315700	-1.61617300	0.07457300	C	4.75872200	-1.75904800	0.06868300
C	3.47107900	-1.73063400	0.03510100	C	3.37397000	-1.83140700	0.02434300
C	2.65259700	-0.58686300	0.01033500	C	2.59376300	-0.66323300	0.01038800
C	3.30695000	0.65895300	0.02834700	C	3.28099700	0.56281800	0.04323300
H	5.14130500	1.73902300	0.08085300	H	5.14713500	1.58572700	0.11129500
H	6.55383300	-0.30297400	0.12178600	H	6.49685600	-0.49761400	0.13369700
H	5.44825200	-2.51719400	0.09182500	H	5.32640100	-2.67747200	0.07775500
H	3.01024800	-2.69916400	0.02399100	H	2.88427200	-2.78533400	0.00178400
H	2.70753400	1.55582900	0.01420600	H	2.71073400	1.47805900	0.03845700
N	1.27007200	-0.55623900	-0.03813400	N	1.20976900	-0.59793200	-0.04170800
C	0.53182600	-1.66386200	0.00446000	C	0.43154000	-1.68206300	0.00038300
O	0.85165700	-2.84340400	0.09016800	O	0.72086300	-2.86691100	0.08846200
C	-1.71807000	-2.40052000	-0.03191300	C	-1.83968300	-2.34215500	-0.03844200
H	-1.51601800	-3.08308700	-0.85411400	H	-1.66574600	-3.02605500	-0.86572800
H	-1.60759200	-2.96275700	0.89270200	H	-1.74288400	-2.91148700	0.88298200
O	-0.79082400	-1.32919200	-0.05766100	O	-0.87393000	-1.30328200	-0.06431200
H	0.42864200	0.80074200	-0.25054900	H	0.52168000	0.66566400	-0.22771000
N	-0.13707900	1.71313000	-0.39127800	N	-0.02206400	1.66736700	-0.38347300
C	-0.08389500	2.53479600	-1.48615000	C	0.07397300	2.47723900	-1.48412500
C	-0.93267600	3.55941600	-1.25743200	C	-0.71465300	3.55346700	-1.27115700
N	-1.48996800	3.34366800	-0.01793300	N	-1.28452900	3.38345400	-0.03131800
C	-0.98945000	2.21331800	0.48547800	C	-0.84591800	2.22847900	0.48127300

H	-1.19270400	4.41464500	-1.84730100	H	-0.92525700	4.41549200	-1.87108100
H	0.54696300	2.32261500	-2.32524500	H	0.69186400	2.22411200	-2.32207000
C	-2.47694400	4.19006600	0.62952000	C	-2.22058300	4.29196300	0.60568600
H	-2.14854800	4.43869000	1.63186000	H	-2.27038700	5.19713900	0.01559200
H	-3.43377800	3.68164400	0.67450700	H	-1.87877100	4.53460000	1.60523200
H	-2.57790800	5.09780700	0.05020600	H	-3.20584500	3.84131300	0.65780600
C	-3.10861300	-1.81642400	-0.14554800	C	-3.20762400	-1.70578300	-0.14105800
H	-3.27098100	-1.12098000	0.67802600	H	-3.25889000	-1.12149800	-1.05969900
H	-3.17559600	-1.23685900	-1.06625000	H	-3.33725400	-1.00713200	0.68552500
C	-4.18844500	-2.89075800	-0.13222100	C	-4.32704600	-2.73864900	-0.12279900
H	-4.10932700	-3.47103800	0.78752900	H	-4.18488400	-3.44045500	-0.94508200
H	-4.01355100	-3.58896300	-0.95125500	H	-4.26342200	-3.32429200	0.79472500
C	-5.59191200	-2.31526900	-0.24862300	C	-5.70823900	-2.10955600	-0.22722600
H	-6.34381500	-3.10173800	-0.23593000	H	-6.48943200	-2.86686400	-0.21127000
H	-5.80782100	-1.63686200	0.57543000	H	-5.89212800	-1.42606800	0.60035200
H	-5.71161400	-1.75706900	-1.17594800	H	-5.81329400	-1.54429500	-1.15202400
C	-1.33169200	1.62681000	1.80066700	C	-1.22659400	1.67977900	1.80344800
H	-1.02391400	2.28863600	2.60670400	H	-0.87355300	2.32496500	2.60468400
H	-2.40450400	1.47348000	1.88348300	H	-2.30711400	1.59999300	1.89050000
H	-0.83008600	0.67385600	1.91311600	H	-0.79162100	0.69564500	1.92528500

1,2-DMI-PC

C	4.61116200	0.61913700	0.08910900
C	5.41155800	-0.51358100	0.14192900
C	4.80808600	-1.76058400	0.12008500
C	3.42897700	-1.89238700	0.04494300
C	2.62699500	-0.75262100	-0.00667900
C	3.23486300	0.50471600	0.01703500
H	5.05766200	1.60074300	0.10597300
H	6.48419700	-0.42431100	0.19936600
H	5.41347500	-2.65255700	0.15933000
H	2.97637800	-2.86456200	0.02823100
H	2.61888800	1.38841100	-0.01833600
N	1.23079000	-0.77911800	-0.09235400
C	0.42718800	-1.86532900	-0.00859700
O	0.77160800	-3.01590200	0.14337600
C	-1.82904400	-2.52859000	-0.04515700
H	-1.64640700	-3.24329900	-0.84231100
H	-1.72789900	-3.04753900	0.90339500
O	-0.85067700	-1.48877900	-0.11632300
H	0.76300600	0.11944700	-0.21530700
N	-0.04049800	1.90831700	-0.43091500
C	0.12752700	2.84488200	-1.41791000
C	-0.64460400	3.92988700	-1.15836000
N	-1.29828000	3.65167100	0.01446400
C	-0.90480000	2.42246400	0.41411300
H	-0.79233500	4.85529100	-1.67901800
H	0.79088100	2.68007400	-2.24586500
C	-2.25942700	4.50391400	0.68175300
H	-1.99664100	4.62017400	1.72767700
H	-3.25907000	4.08595700	0.61016500
H	-2.24878800	5.47662200	0.20670200
C	-3.18984900	-1.88741000	-0.18049300
H	-3.31616800	-1.15090600	0.61247000
H	-3.23704400	-1.34875200	-1.12630000
C	-4.31172400	-2.91604900	-0.11351700
H	-4.17240600	-3.65509100	-0.90277100
H	-4.24887700	-3.45811000	0.83026000

**C**	**-5.68998800**	**-2.28610100**	**-0.24587500**
**H**	**-5.79313300**	**-1.76309300**	**-1.19530500**
**H**	**-6.47367900**	**-3.03913700**	**-0.19513100**
**H**	**-5.87023800**	**-1.56529600**	**0.55011000**
**C**	**-1.40362000**	**1.77098100**	**1.65328600**
**H**	**-1.10010300**	**2.32039000**	**2.54265600**
**H**	**-2.49004000**	**1.71113400**	**1.66083200**
**H**	**-1.00299100**	**0.76580700**	**1.71439800**
